# Impact of molecular profiling in patients with endometrial cancer: real world experience from India

**DOI:** 10.3389/fonc.2026.1923324

**Published:** 2026-09-15

**Authors:** Richa Kaushik, Basumita Chakraborti, Anik Ghosh, Santam Chakraborty, Tapesh Bhattacharyya, Jagannath Mishra, Subhashree Rout, Divya Midha, Deepak Mishra, Sushant Vinarkar, Somnath Roy, Oindrila Roy Chowdhury, Jaydip Bhaumik

**Affiliations:** 1 Department of Gynecological Oncosurgery, Tata Medical Centre, Kolkata, India; 2 Department of Radiation Oncology, Tata Medical Centre, Kolkata, India; 3 Department of Pathology, Tata Medical Centre, Kolkata, India; 4 Department of Molecular Pathology, Tata Medical Centre, Kolkata, India; 5 Department of Medical Oncology, Tata Medical Centre, Kolkata, India

**Keywords:** adjuvant treatment, endometrial cancer, India, molecular profiling, real world

## Abstract

**Objective:**

The introduction of The Cancer Genome Atlas (TCGA) had made a paradigm shift towards molecular based classification that impacted the treatment of endometrial cancer (EC). Here we aim to share our initial experience with the implementation of molecular testing into daily clinical practice of endometrial cancer.

**Methods:**

This is an ambispective cohort study on prospectively collected data of 350 endometrial cancer patients who underwent primary surgery between 2020 and 2024 at our institution. Clinical, radiological, pathological and treatment related data were obtained through hospital management systems (HMS).

**Results:**

The median age of our cohort was 59 years. Most of the patients had endometrioid histology (267, 76.3%) and early-stage disease (288, 82.3%). Effectively 64 patients had p53 mutation if 8 double classifiers with p53 abnormality were excluded.115 patients had mismatch repair deficient (MMRd) tumors. Out of 350 patients POLE mutation was tested in 110 (31.4%). 5 patients out of these 110 (4.5%) had pathogenic POLE mutations and one had POLE variant of unknown significance (VUS). None of the patients with POLE ultra-mutated tumors received any adjuvant treatment or developed any recurrences. All of 11/64 p53 mutant patients (17.2%) developed systemic metastatic recurrences. At a median follow-up of 33 months, 3-year overall survival (OS) of POLE ultra-mutated and p53abn subgroups were 100% and 87% respectively. MMRd and nonspecific molecular pattern (NSMP) subgroups achieved OS of 97% and 96% respectively.

**Conclusion:**

Assessment of the molecular profile is currently a standard investigation for evaluation of EC at our institution. This study represents a real-world experience of routine molecular marker testing among patients with EC from a tertiary care center of India.

**Synopsis:**

We aim to share our initial experience with the implementation of molecular testing into daily clinical practice of endometrial cancer. Phase wise implementation of molecular testing started at our institute in 2015.It became routine in 2023. POLE ultra-mutated tumors have shown excellent prognosis. None of those patients developed any recurrences even without any adjuvant treatment. p53 abnormal tumors have the worst prognosis, with systemic recurrence being the predominant pattern of failure. MMRd and NSMP subset of patients performed moderately in between POLE mutant and p53 abnormal tumors.

Molecular profiling is the most important futuristic step towards optimized adjuvant treatment for endometrial cancer in a developing country like India.

## 1 Introduction

A majority of the patients of endometrial cancer (EC) present in early stage with low grade histology (Grade 1 and 2 endometrioid type). Around 85% of the EC are of endometrioid histology with favorable prognosis and achieve a 5 year survival of around 95% (1). The standard treatment is total hysterectomy with bilateral salpingo-oophorectomy and lymph node assessment and dissection in patients with substantial risk of lymph node metastasis (2).

The introduction of molecular classification by The Cancer Genome Atlas (TCGA) has initiated a shift towards molecular classification based prognostication that created an impact on the treatment of endometrial cancer (3).The molecular classification integrated into clinicopathological features of EC has put forward four different molecular groups with distinct tumor biology and prognosis. EC with a pathogenic mutation of DNA polymerase epsilon gene (POLEmut) has an excellent prognosis, and, on the other hand, patients with TP53 abnormality have the worst prognosis. Mismatch repair deficient (MMRd) EC has intermediate prognosis, whereas EC with no specific molecular profile (NSMP) has a stage-dependent prognosis (4–7). Several studies have demonstrated the independent prognostic impact of molecular classification on EC treatment outcome (8).Many international guidelines and consensus recommendations have already incorporated molecular classification in risk stratification of EC which has also guided escalation and de-escalation of adjuvant treatment in different risk groups (9).Several studies on POLE mutant patients demonstrated almost no locoregional recurrences or systemic relapses in 5 years with or without adjuvant treatment and the data is still accumulating. On the contrary, stage I-IV EC with p53 abnormality is considered as high risk and requires escalated treatment with adjuvant chemotherapy and radiotherapy. The PORTEC 3 trial molecular data has shown an inferior outcome with addition of chemotherapy in MMRd patients in advanced stage endometrial cancer and warrants the role of immunotherapy (8).

Though ESGO-ESTRO-ESP consensus guidelines (9) and FIGO 2023 recommend integration of molecular classification in risk stratification and staging, very few centers in India have incorporated molecular classification in their routine clinical practice to decide adjuvant treatment. Even among the centers in India who are integrating molecular classification in risk stratification and deciding adjuvant treatment, a lot of heterogeneity exists in terms of methodology and implementation.

Here we investigate the initial outcome of all four molecular classes of EC in a real world scenario of a low middle income group (LMIG) country like India.

## 2 Methods

This is an ambispective cohort study of 350 EC patients who underwent primary surgery between 2020 and 2024 at Tata Medical Center, Kolkata. Patients who underwent upfront radical surgery for non-metastatic disease between January 2020 and August 2022 were identified retrospectively from institutional records, subsequently following the initiation of the prospective study phase in September 2022, consecutive eligible patients were enrolled prospectively until June 2024 using the same predefined eligibility criteria and data collection protocol. Patients who underwent radical surgery at an outside center and came to us only for adjuvant treatment were excluded from this analysis as the quality and completeness of surgery will be heterogeneous in that subset of the patients. All pathology slides were assessed by experienced senior onco-pathologists and molecular biologists. Prospectively collected clinical and radiological data, medical history, and treatment data as well as pathology-associated parameters were obtained through a review of digital medical records and pathology reports in hospital management systems (HMS). During follow up all these patients underwent clinical examination every 3 monthly for 1st 2 years, 6 monthly from 3rd to 5th year and annually thereafter. During these periods they also underwent symptom directed imaging in the form of CECT Thorax and abdomen and MRI Pelvis as and when required depending on symptoms. Follow-up data on recurrence and survival were available through HMS standardized databases.

Recurrence was defined as the appearance of radiological or pathologically confirmed endometrial cancer after completion of upfront surgery with or without adjuvant radiotherapy, chemotherapy, or both in patients who had no evidence of residual disease at the end of treatment. The date of recurrence was defined as the date on which recurrence was first confirmed.

Patients who remained alive without any event at the time of last available follow up were censored on the date of their last disease assessment. Patients who were lost to follow up were also censored at the date of their last documented clinical evaluation at which they were known to be event free. Information regarding death was obtained from hospital medical records, follow up clinic visits, discharge summaries and direct communication with patients or their family members by telephone, where applicable. The cause of death was determined based on the available clinical documentation and was categorized as disease related or non-disease related whenever sufficient information was available. This study protocol was reviewed and approved by the institutional review board (TMC-IRB).

### 2.1 Molecular classification

Since 2015, we have implemented molecular profiling in phases. By 2020, we conducted immunohistochemistry (IHC) for p53 and MMR proteins (MLH1, MSH2, MSH6, PMS2) in nearly all endometrial cancer (EC) patients. Detection of MMRd is carried out through IHC for MMR proteins or through MSI analysis by polymerase chain reaction (PCR). MMR IHC simply detects the presence or absence of the 4 MMR proteins within cancer cells. MMR IHC is straightforward to interpret in most cases based on the fact that MMR proteins function in dimers: loss of MLH1 nuclear expression is usually accompanied by PMS2 loss, whereas MSH2 loss is accompanied by MSH6 loss in the context of satisfactory internal positive control (lymphocytes, endometrial stromal cells). MSI testing detects differences in the lengths of a selection of microsatellites, resulting from insertion-deletion errors, between normal cells and tumor cells. In case of loss of nuclear staining of MLH1 and PMS2, testing for MLH1 promoter hypermethylation is done by methylation-specific PCR. MMR deficiency (MMRd) was identified by the loss of nuclear staining in any of the four MMR proteins. p53 IHC is interpreted as abnormal and wild-type staining patterns. Normal (wild-type) expression is characterized by a heterogeneous staining pattern in which there are variable number of positive cells with variable staining intensity admixed with negative cells. Three abnormal or mutation-type IHC patterns of p53 expression have been described which predict underlying TP53 gene mutations with 96% sensitivity and 100% specificity. Overexpression type (nuclear overexpression in 80% to 100% of cells) is associated with nonsynonymous missense mutations. Complete absence (null pattern) is associated with loss-of-function mutations. Cytoplasmic expression (associated with loss-of function mutation disrupting nuclear localization domain) seen rarely. Rarely truncating or splice site mutations can result in wild-type expression (because of a non-functional p53 protein). These markers are assessed in initial endometrial biopsy specimens currently.

In late 2023, POLE mutation testing became routine, using Sanger sequencing on postoperative specimens. The POLE exonuclease domain (Exons 9-14) was analyzed. DNA was extracted from fresh frozen paraffin-embedded tumor blocks. PCR and sequencing were performed using specific primers, with results analyzed for nucleotide changes as reported by Yu et al. (10). If molecular features (p53, MMR, POLE) were undetermined, the case was classified as EC with no specific molecular profile (NSMP). Tumors with multiple molecular features were considered multiple classifiers. ECs with a pathogenic POLE mutation and p53 abnormal expression and/or MMR protein loss were classified as POLE mut EC. ECs with MMR protein loss or MSI-high and p53 mutant staining were classified as MMRd. Patients were categorized into prognostic risk groups per the 2021 ESGO/ESTRO/ESP classification, based on FIGO 2009 stage, histology, grading, lympho-vascular space invasion (LVSI), and molecular subtypes (POLEmut, p53abn, MMRd, NSMP). Our institutional molecular subtyping workflow is displayed in **Figure 1**.

**Figure 1:**
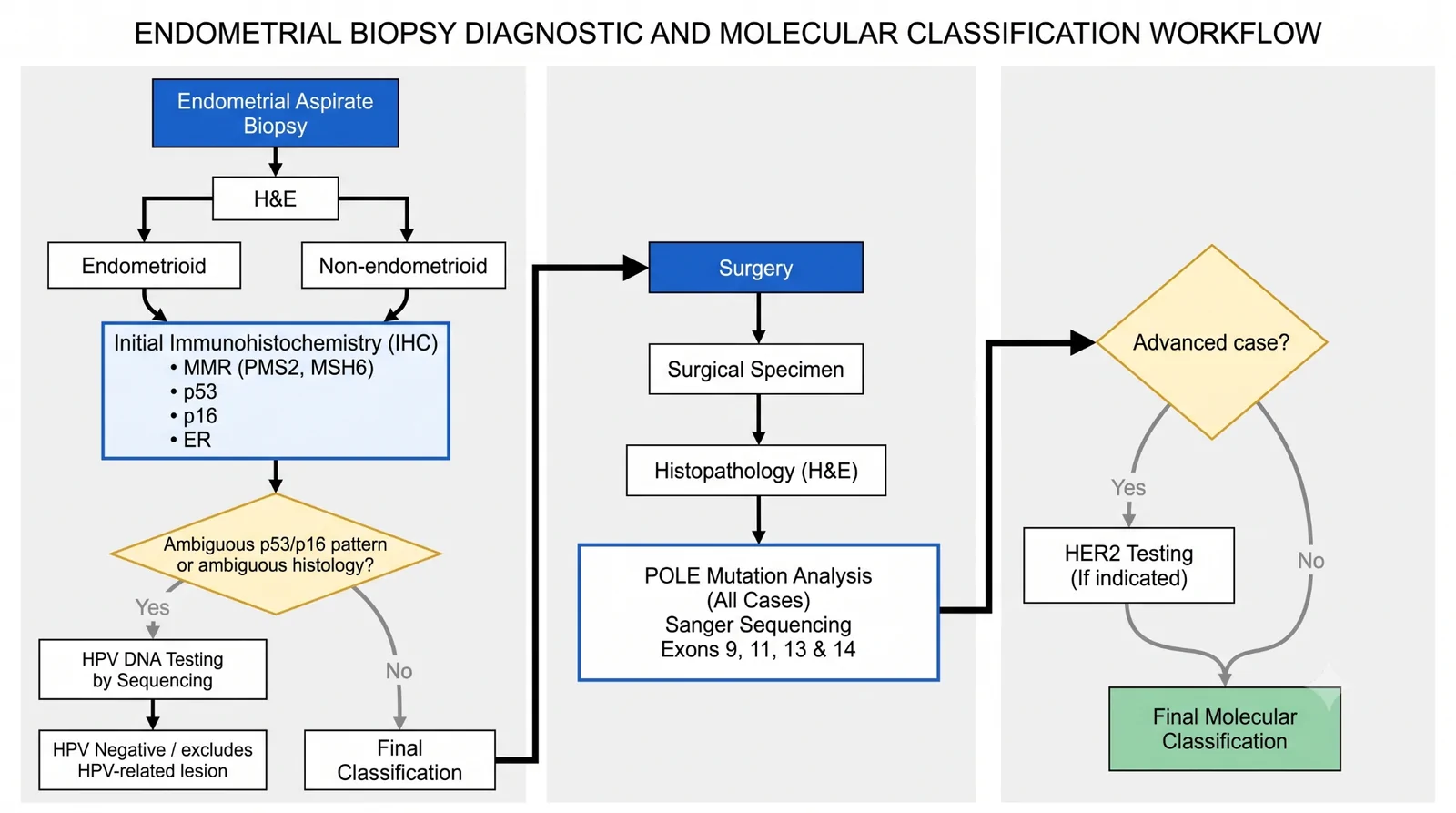
Institutional molecular subtyping workflow.

### 2.2 Statistical analysis

Statistical analysis was conducted using R software version 4.4.2. Categorical variables were reported as frequencies and percentages; continuous variables as means and standard deviations or medians and range. Survival curves were created using the Kaplan Meier method, with comparisons via log-rank test. Univariable Cox regression analysis assessed recurrence or death risk factors. As the number of events was small (23 recurrences) and the POLEmut subgroup had no events Cox proportional hazard modelling with multiple covariates was not considered. A p-value < 0.05 was deemed statistically significant.

## 3 Results

### 3.1 Baseline demographics

The median age of this cohort was 59 years (range 54–56 years). 77.1% of our patients had endometrioid histology. Only 21.1% of the patients had substantial lymphovascular space invasion in the final histopathology report. A total of 82% of the patients had early stage (stage I and II) disease. Effectively 64 patients had p53 abnormal tumors out of which 4 had p53 null type (8 double classifiers, who had p53 abnormality along with MMRd or POLE mutant were MMRd or POLEmut tumors). Demographic profile and clinicopathological features are displayed in **Table 1**.

**Table 1:** Demographic profile and clinicopathological features(n=350).

Characteristics	Number (%)
Age	Median 59 years (54–66 years)
Body Mass Index (BMI)	Median 29.5kg/m^2^ (26.3-32.5)
Comorbidities	
Hypertension	222(63.4%)
Diabetes	126(36%)
Hypothyroidism	114(32.6%)
Histological type	
Endometrioid	270 (77.1%)
Non endometrioid	80 (22.9%)
Grade	
Low grade (Endometrioid G1and G2)	235 (67.1%)
High grade (Endometrioid G3 and Non Endometrioid)	115 (32.9%)
LVSI	
Focal/Negative	276 (78.9%)
Substantial	74 (21.1%)
Myometrial invasion	
No	31 (8.9%)
Less than 50%	164 (46.9%)
More than 50%	155 (44.2%)
FIGO Stage	
Stage IA	185 (52.9%)
Stage IB	84 (24%)
Stage II	19 (5.4%)
Stage III and IV	62 (17.7%)
Molecular subtypes	
POLE mutant (110 tested)	5(4.5%)
MMR deficient (350 tested)	115(32.9%)
p53 abnormal (305 tested)	64(20.9%)
NSMP (110 tested)	51(46.4%)
Molecularly unclassified (350 tested)	115(32.9%)
Pelvic lymph node assessment	
Median number of pelvic lymph node dissected	8 (range 1-24)
Median number of positive pelvic lymph node	0 (range 0-8)
Adjuvant treatment	
Adjuvant radiation and or chemotherapy	169(48.3%)
No adjuvant treatment	181(51.7%)

### 3.2 Timeline of molecular testing

Molecular profiling of EC was introduced in our Institute through multidisciplinary tumor board (MDT) discussion in a phased manner (**Figure 2**). During 2015 and 2016 it was done for the EC patients younger than 50 years of age and then extended to till 60 years. Soon, as the world literature suggested universal MMR testing as the means to detect Lynch Syndrome, our MDT moved in that direction and by 2020 we were doing MMR-IHC for all EC patients. From late 2023 POLE mutational profiling became routine for EC patients in our Institute and currently this is routinely undertaken for all patients with EC.

**Figure 2:**
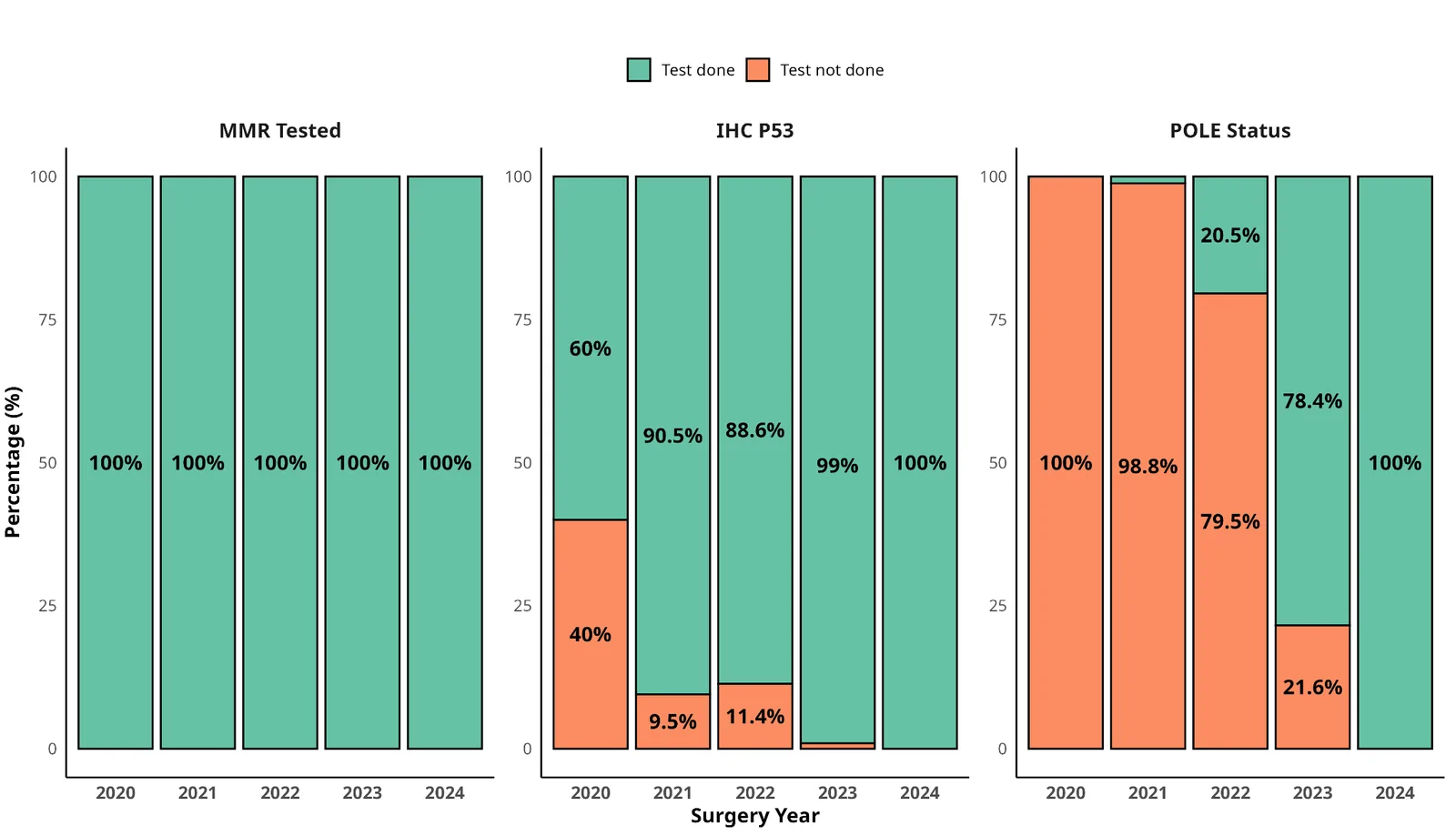
The stacked bar plot showing timelines of molecular testings.

Microsatellite instability through MMR-IHC has been tested in all operated endometrial cancer cases (100%) since 2020. p53 was tested in only 60 percent of cases in 2020, gradually it increased to almost 90% in 2021 and 2022 and reached 99% and 100% in 2023 and 2024 respectively. Overall p53 was tested in 305 patients in this cohort (305/350; 87.1%). POLE mutation testing by Sanger sequencing was checked in only 1.2% of cases in 2021, it reached 20.5% in 2022 and increased to 78.4% in 2023 and 100% in 2024. Out of 350 patients, POLE mutation was tested in 110 (31.4%) patients.

In 240 patients (68.6%) POLE testing was not performed. Finally we found 115 MMRd patients, effectively 64 p53 abnormal patients, 5 patients had POLE mutation,51 patients had NSMP and 115 patients were considered as molecularly unclassified.

### 3.3 POLE mutant group

A total of 5 patients (5/110; 4.5%, 95%CI 1.95 - 10.2%) harboring pathogenic POLE mutations (POLEmut) were identified in this institutional cohort. One patient had a POLE variant of unknown significance (VUS). All POLE mutations were located in Exon 9 locus of the POLE gene, whereas the POLE VUS was detected in Exon 26. All POLEmut patients had endometrioid histology and belonged to stage I disease except one, who had stage II disease. Two out of five patients had grade 3 endometrioid histology. The POLE Sanger sequencing assay detected the POLE variant: p.Pro286Arg (c.857C>G; exon 9) in all 5 POLEmut cases. Two of the POLEmut cases exhibited ‘multiple classifier’ features overlapping with MMRd and p53 abnormal cases respectively; however, both were designated POLEmut in the final classification. According to the MDT consensus adjuvant treatment deescalation was done in 3 patients because of POLE positivity. After MDT discussion none of the POLE mut patients received any adjuvant treatment and were kept on follow up. All of those patients are still on follow up and continue to be free of any locoregional or systemic recurrence. The clinicopathological characteristics, de-escalation of adjuvant treatment and current status of POLE ultra mutated patients are displayed in **Table 2**.

**Table 2:** POLE positive patient characteristics.

Patients	Histology	Stage and grade	Mutation	Adjuvant treatment	MDT decision change	Status at last follow up
Case 1	Endometrioid	IAG2	Exon 9	No	No	Disease free, Alive
Case 2	Endometrioid	IBG2	Exon 9	No	Yes	Disease free, Alive
Case 3	Endometrioid	IIG3	Exon 9	No	Yes	Disease free, Alive
Case 4	Endometrioid	IAG1	Exon 9	No	No	Disease free, Alive
Case 5	Endometrioid	IBG3	Exon 9	No	Yes	Disease free, Alive
Case 6	Endometrioid	IAG2	VUS Exon 26	No	No	Disease free, Alive

### 3.4 Double classifiers

In this cohort, overall 9 patients had tumors with double molecular aberrations. Out of these 9 double classifiers,7 had MMRd and p53 overlap and they were considered MMRd tumors. Two POLEmut cases had overlap, one with p53 mutation and another MMRd tumor respectively. These two cases were considered as POLEmut tumors and they did not receive any adjuvant treatment as other pure POLE ultra-mutated cases. All double classifiers are alive at 3 years of follow up. The Venn diagram showing the double classifier is displayed in **Figure 3**.

**Figure 3:**
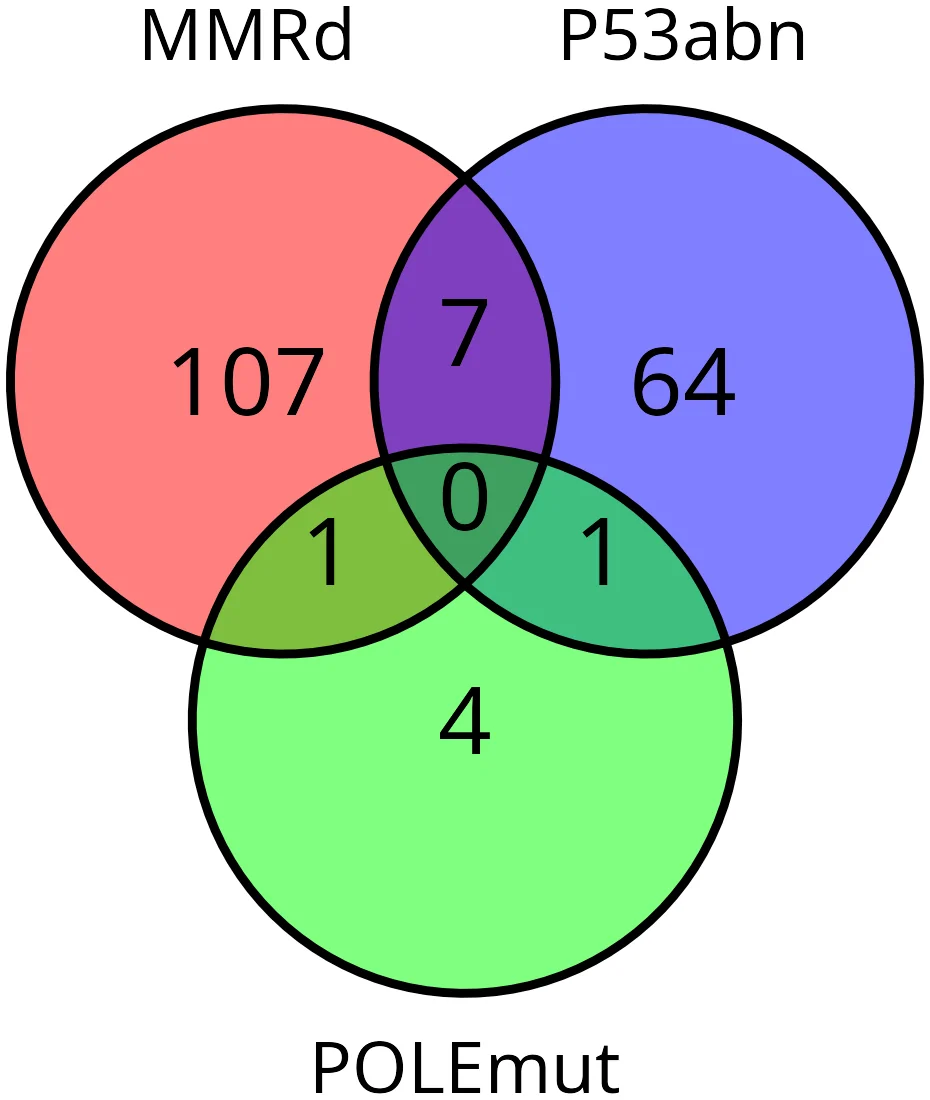
Venn diagram showing the double classifier.

### 3.5 Pattern of recurrence according to molecular profile

In total, out of the 350 women, 23 (6.57%, 95%CI 4.42 - 9.67%) had recurrences, out of which systemic metastasis were seen in 20 cases and only 3 patients had locoregional recurrences. Out of 20 distant metastases, 12 patients had abdominal metastasis, 7 patients had extra abdominal metastasis and one patient had both abdominal and extra abdominal metastasis. None of the POLE ultra mutated tumors developed any recurrences. Out of 64 p53 mutant patients, 11 patients (17.2%) developed recurrences which are all systemic metastasis. Out of these 11 recurrent p53 mutant patients abdominal recurrences occurred in 6 patients (55%). None of the p53 mutated patients developed locoregional failure. All p53 mutant recurrences had non endometrioid histology and 9 out of 11 recurrences had advanced stage disease at the time of diagnosis. Out of 115 MMRd patients 7 patients (6.1%, 95%CI 2.98 - 12.03) developed recurrence, out of which 4 patients (57.1%) developed abdominal recurrences, not much different from p53 mutant patients. One patient developed local or locoregional failure in the MMRd group. Out of 51 NSMP patients only 2 patients (3.9%) developed recurrences, out of which one patient failed systemically and another one failed locally. From 18 months onwards the p53 subgroup surpassed other subgroups in terms of relapse as shown in **Figure 4**. Patterns of recurrences according to molecular profiling are shown in **Table 3**.

**Figure 4:**
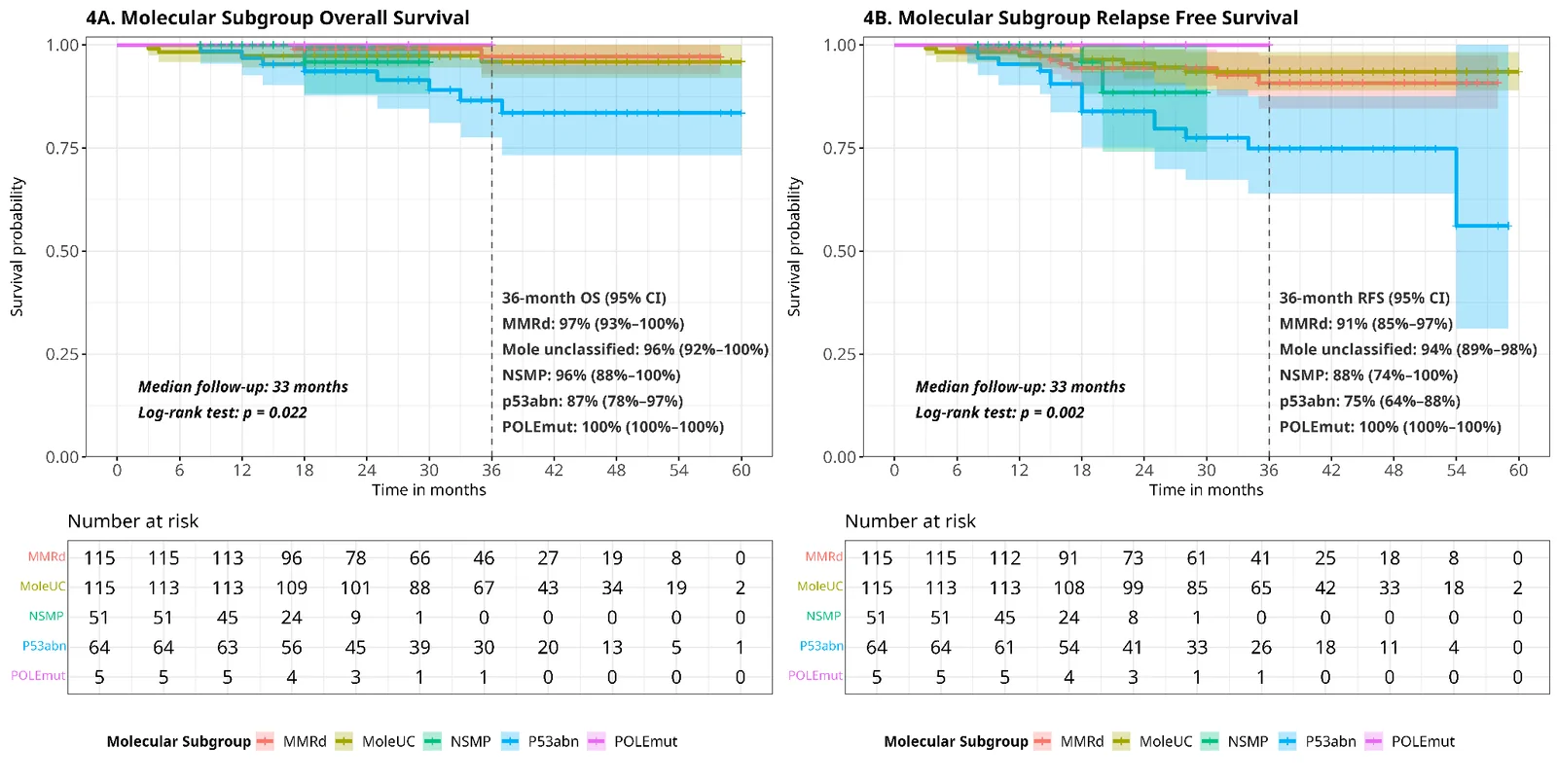
**(A)** Molecular subgroup wise Overall Survival, **(B)** Molecular subgroup wise Relapse free survival.

**Table 3:** Molecular classification wise pattern of recurrence.

Pattern of recurrence	Overall N = 350	MMRd N = 115	Molecularly unclassified N = 115	NSMP N = 51	p53abn N = 64	POLEmut N = 5
Pattern						
Abdominal	12(3.4%)	4 (3.5%)	2 (1.7%)	0 (0%)	6 (9.4%)	0 (0%)
Abdominal + Extraabadominal	1 (0.3%)	0 (0%)	0 (0%)	0 (0%)	1 (1.6%)	0 (0%)
Extraabdominal	7 (2.0%)	1 (0.9%)	1 (0.9%)	1 (2.0%)	4 (6.3%)	0 (0%)
Locoregional	3 (0.9%)	1 (0.9%)	1 (0.9%)	1 (0%)	0 (0%)	0 (0%)

### 3.6 Survival analysis according to molecular profile

At a median follow-up of 33 months, the 3 year OS of POLEmut and p53abn subgroups were 100% and 87% respectively. MMRd and NSMP subgroups achieved OS in between POLE ultramutated and p53 abn subgroups and they were 97% and 96% respectively. Similarly POLEmut and p53abn subgroups achieved a 3 year RFS of 100% and 75% respectively. MMRd and NSMP subgroups achieved a modest 3 year RFS of 91% and 81% respectively. The OS and RFS of different molecular subgroups are shown in **Figures 4A, B**.

Apart from molecular subgroup wise analysis, as we also attempted to evaluate the impact of stage on outcome, it is also noteworthy to mention that the 3year RFS of patients with stage I-II was 92% (95% CI 88%-96%) vs 74% (95% CI 64%-87%) for stage III-IV disease(p=0.00017) (**Figure 5A**). The 3 year OS for patients with stage I-II disease was 96% (95% CI-93%-99%) vs 88% (95% CI 80%-98%) for stage III-IV disease(p=0.039)(**Figure 5B**).

**Figure 5:**
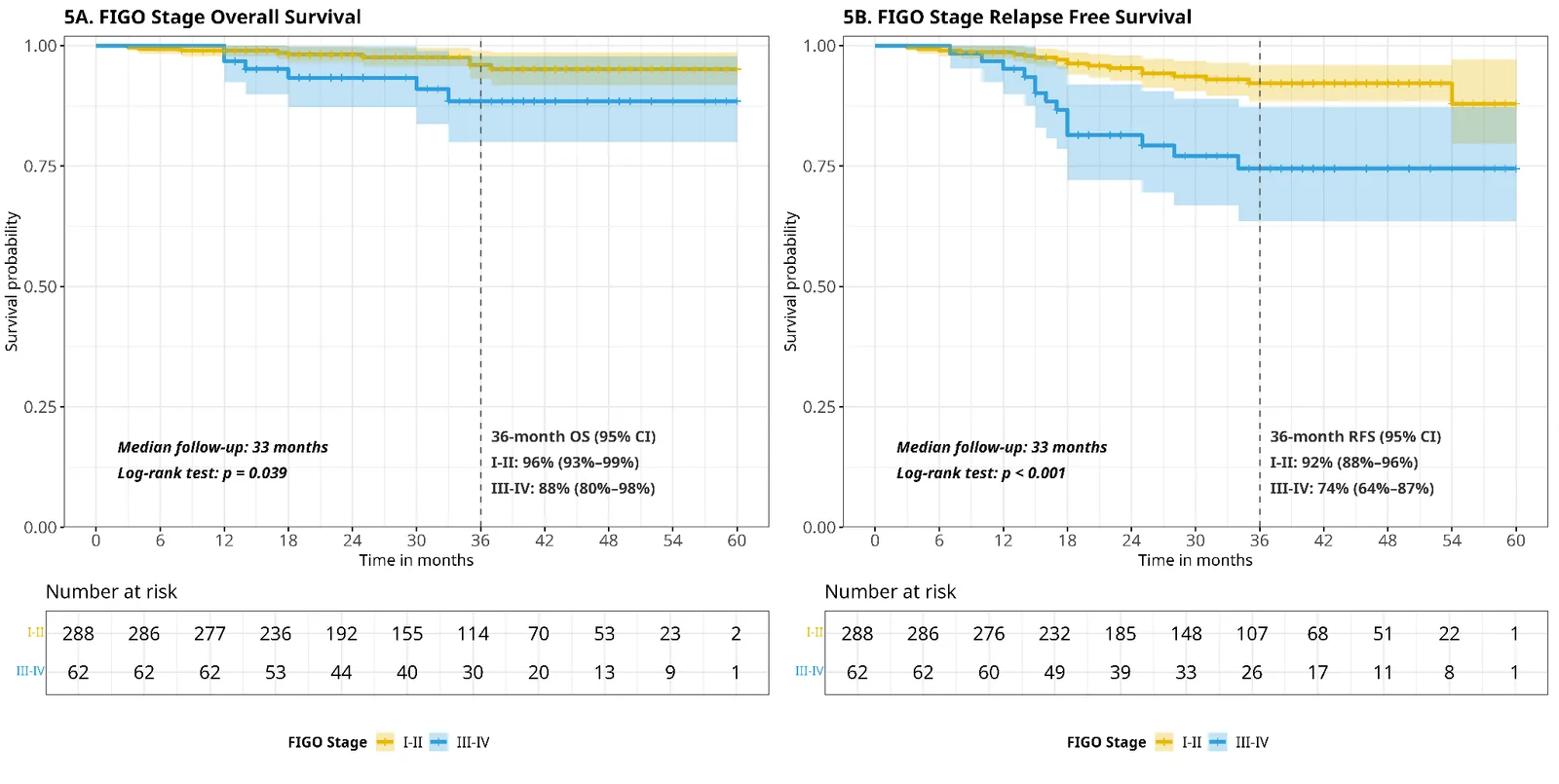
**(A)** FIGO stage wise Overall Survival. **(B)** FIGO stage wise Relapse free survival

### 3.7 Subset analysis according to molecular profile

On subset analysis, all molecular subgroups had endometrioid histology in majority, except p53abn subgroups, where 84.3% patients had a non-endometrioid histology. Similarly, the majority of the patients had grade 1 and grade 2 endometrioid carcinoma in all molecular subgroups except p53 abnormal subgroup, where 95.3% patients had high grade endometrioid or non-endometrioid carcinomas. All molecular subgroups had early stage disease except p53, where 43.7% patients had advanced stage III and IV disease. Substantial LVSI was also highest (37.5%) in the p53 abnormal subgroup. Depth of myometrial invasion was not significantly different among different subgroups. Subset analysis according to molecular profile is displayed in **Table 4**.

**Table 4:** Subset analysis according to molecular profile.

Characteristics	Overall n=350	MMRd n=115	NSMP n=51	P53abn n=64	POLEmut n=5	Molecularly unclassified (n=115)
Histological type						
**Endometrioid**	270 (77.1%)	100 (88.0%)	49 (96.0%)	10 (15.6%)	5 (100%)	106(92.2%)
**Non Endometrioid**	80 (22.9%)	15 (12.0%)	2 (4.0%)	54 (84.3%)	0 (0%)	9(8.8%)
Grade						
**High grade (Endometrioid Grade 3 and Non endometrioid)**	115(32.9%)	28 (24.3%)	4 (7.8%)	61 (95.3%)	2 (40%)	20(17.4%)
**Low grade (Endometrioid Grade 1 and 2)**	235 (67.1%)	87 (75.6%)	47 (92.2%)	3 (4.7%)	3 (60%)	95(82.6%)
FIGO stage						
**Stage I and II**	288 (82.2%)	100 (87%)	47 (92.2%)	36 (56.3%)	5 (100%)	100(87%)
**Stage III and IV**	62 (17.7%)	15 (13.0%)	4 (7.8%)	28 (43.7%)	0 (0%)	15(13%)
LVSI						
**Negative/Focal**	276 (78.9%)	88 (76.5%)	46 (90.2%)	40(62.5%) 24	4(80.0%)	98(85.2%)
**Substantial**	74 (21.1%)	27 (23.5%)	5 (9.8%)	(37.5%)	1(20.0%)	17(14.8%)
Myometrial invasion						
**No**	31 (8.9%)	10 (8.7%)	4 (7.8%)	8 (12.5%)	0 (0%)	9(7.8%)
**Inner half**	164 (46.8%)	54 (46.9%)	27 (52.9%)	21 (32.8%)	2 (40%)	60(52.2%)
**Outer half**	155 (44.3%)	51 (44.3%)	20 (39.2%)	34 (53.1%)	3 (60%)	46(40%)

## 4 Discussion

The last decade has seen a paradigm shift in the diagnosis of endometrial carcinoma and the classification has evolved from a dualistic clinico-pathological one to a histotype-based classification and more recently to a prognostically significant non-overlapping molecular subtyping (11, 12).To the best of our knowledge this is the first study from India supporting the feasibility and describes the real world experience in patients of EC treated with protocol driven need based surgery with or without adjuvant treatment. The present study showed that POLE ultra mutated EC have excellent outcomes even without receiving any adjuvant treatment. Out of the 5 POLEmut patients, 2 belonged to high intermediate risk (stage II G3 and IB G3) and one patient belonged to intermediate risk group (IB, G2) according to ESGO-ESTRO-ESP consensus guideline. Our MDT decided for de-escalation of adjuvant treatment in those patients with no adjuvant radiotherapy. At a median follow up of 33 months, none of the patients recurred including a patient who had a p53 co-mutation and we could achieve a 3-year OS and RFS of 100%. Our findings are consistent with previously published data demonstrating the excellent prognosis associated with POLE mutant patients, while acknowledging the larger studies are required before drawing some firm conclusions regarding treatment deescalation (13).

Horeweg et al. suggested that even in the absence of adjuvant treatment, the risk of recurrence seems to be very low, as shown in 21 women participating in the PORTEC-1 trial and previously in 26 women with high-grade POLEmut EC included in a Danish study (14). The POLE BLUE trial within the RAINBO program is prospectively investigating the deescalation of adjuvant treatment for POLEmut EC, with no adjuvant treatment for early stages and probably de-escalated treatment for stage III disease (15). The ongoing PORTEC 4a study will compare standard adjuvant brachytherapy to individualized treatment based on the tumor’s molecular profile, including observation after surgery for women with POLEmut EC (16).

One of the key hurdles for implementing POLE testing for endometrial cancer was its high cost, especially in a setup where outpatient investigations are not insurance-covered for most of the patients. While Next Generation Sequencing (NGS) is considered the gold standard for comprehensive POLE mutation detection, it is expensive to implement. Hence we started using a low-cost Sanger sequencing technique for POLE mutation covering Exon 9–14 only as a pilot study. Between 2021-22, POLE mutations were requested on a case-to-case basis in a MDT after discussing with the pathologist. Slowly as evidence emerged about the good outcomes associated with POLE testing internationally, the MDT decided to adopt POLE testing for all patients in the year 2024 and none of our patients missed whole molecular profiling in the same year However, to optimize the cost, turnaround time and to mitigate POLE testing failure rates due to poor DNA from pre-operative biopsy, it was decided that the POLE testing would be ordered in the postoperative specimen instead of the pre-operative specimens, as insurance coverage of investigations ordered during in-patient admissions was possible. At the same time, a government-sponsored health insurance scheme became available to a significant proportion of the patient population, which covered investigations in the inpatient settings.

However, in resource constrained settings especially in LMICs a more targeted testing strategy can be adopted by omitting testing in patients with low-risk disease, where adjuvant treatment is not routinely offered. Additionally, the current evidence base is not sufficient to support treatment de-escalation based on POLE testing for patients with advanced or node-positive disease. In the current study 17% (62/350) of the patients would fit these criteria in view of the advanced stage. At the same time, it is important to appreciate the emerging body of evidence regarding the prognostic impact of POLE mutations has prompted the inclusion of node-negative, non-endometroid histologist with uterus confined disease into the low-risk disease in the ESGO-ESTRO-ESP consensus guidelines (17). Alternatively, more pocket friendly POLE mutation testing approaches should be explored. These will include strategies like using qPCR-based hotspot and use of artificial intelligence for the prediction of molecular classes on hematoxylin-eosin-stained whole slide images (18, 19).

The poor prognosis of the p53abn group observed in our cohort could be attributed to its high rate of distant recurrences, which are associated with the worst survival outcomes[8]. This subgroup had a 3 year OS and RFS of 87% and 75% respectively as compared to 5 year PFS of around 50% as shown in PORTEC and other studies. Apart from the intrinsic aggressive biology of the p53 subgroup the possible hypothesis of the worst outcome of p53abn patients is substantiated by the fact that 84% patients of this subgroup had non endometrioid histology, 95% had high grade disease and also had a relatively higher proportion of advanced stage disease. Systemic metastasis is the only form of relapse in this subgroup which warrants intensification of further systemic treatment along with optimal locoregional treatment.

In this study MMR deficient subset of patients achieved a 3 year OS and PFS of 97% and 91% respectively. Out of the 115 MMRd patients, only one developed locoregional failure and five developed systemic metastasis of which 4 were of abdominal relapses. Siegenthaler et al. demonstrated distinct patterns of recurrence according to molecular subgroups, with more locoregional recurrences in the MMRd group (20).In the study of Kim et al., MMRd EC showed a unique recurrence pattern involving retroperitoneal lymph nodes, compared to more distant recurrences in the MMRp group (21). As this subset of patients have a relatively low risk of locoregional recurrence, the absolute benefit of EBRT might not surpass the risk of toxicity as shown by Nout et al (22, 23). In early-stage MMRd EC, substantial LVSI is associated with a significantly higher risk of recurrence and death (5) and should be considered as one of the important deciding factor for adjuvant radiotherapy. The PORTEC-4a trial currently investigates whether MMRd EC with substantial LVSI benefit more from EBRT than standard VBT (16). Further research to identify subgroups within stage I endometrioid MMRd EC that would benefit from EBRT is of interest; the updated results of PORTEC 4a recently discussed in ESTRO 2025 will answer many such questions.

Out of 51 NSMP patients only one patient had vaginal recurrence and another one had an extraabdominal relapse. None of our patients suffered from regional relapse. Aznar et al. showed that NSMP patients experienced distant failures in a higher proportion (10.3% vs 6.4%, p<0.001) as compared to MMRd patients. These findings are consistent with those studies published by Stelloo et al. (5) reporting 39% of distant failures for NSMP tumors, and with those of Siegenthaler et al. (20) and Luzarraga et al (24). However, the NSMP group exhibits a variable prognosis, and new molecular factors, such as the presence of L1CAM or the absence of estrogen receptors, have been recently described as predictors of worst prognosis (25, 26).In our cohort 51 NSMP patients 89% had estrogen receptor (ER) receptor positivity and progesterone receptor (PR) was positive in 4 patients (8%)only.L1CAM was not performed in any of the cases.

It is also interesting to note that we have recognized 8 patients younger than 40 years of age who were operated outside during this time span and were not part of this analysis. Out of these 8 patients 2 were MMR deficient (25%), 1patient (12.5%) had p53 mutation and none of the patients was POLE mutated.

Resource limitations, including the high costs of IHC reagents, limited access to automated staining platforms, shortages of experienced gynecologic pathologists were also the major hurdles in adoption of p53 and MMR testing as a routine clinical practice. Variability in tissue processing, lack of standardized reporting and limited stringent quality assurance program might further affect the diagnostic accuracy. Moreover, confirmatory molecular investigations such as MLH1 promoter hypermethylation and POLE sequencing were frequently unavailable restricting complete molecular classification.

Despite these challenges, we adopted a pragmatic, phased implementation strategy which facilitated the gradual integration of molecular testing into routine clinical practice. p53 and MMR IHC were considered as the initial priority, as these relatively affordable assays identify most clinically relevant molecular subgroups and had immediate implications for prognosis and adjuvant treatment selection. More advanced molecular tests including MLH1 hypermethylation analysis and POLE sequencing were subsequently adopted to optimize resource utilization and ensure quality. Concurrent investments in training, standardized laboratory protocols, quality assurance programs helped us to maintain diagnostic consistency. Gradually National guidelines and reimbursement policies are being progressively incorporated to promote equitable access to molecular stratification in endometrial cancer. Such a stepwise approach provided a feasible pathway for LMIC like us to adopt evidence based molecular risk stratification while minimizing costs and maximizing clinical impact. Future nationwide collaborative research should also evaluate cost effectiveness, phase wise implementation outcomes and long-term impact of phase wise implementation of molecular testing strategies in extremely diverse population of our country.

### 4.1 Limitations

The major strength of the study is reasonably good sample size, data for analysis collected in electronic medical records and stringent follow up.

However the study is not without its limitations. It is an ambispective single-center study. POLE mutation was tested in only 31.4% of our cohort. Sanger sequencing was restricted to exons 9-14.Consequently, pathogenic variants located outside these sequenced regions could not be detected. Although exons 9–14 encompass the majority of clinically relevant hotspot mutations for the gene under investigation, rare pathogenic variants in other exons or non-coding regulatory regions might have been missed. Therefore, a small proportion of patients may have been classified as wild type. Comprehensive approaches such as full genome sequencing or next generation(NGS)panels would provide more complete mutational profiling and may identify additionally clinically relevant variants. A significant proportion of patients (32.9%) were considered as molecularly unclassified. A very small number of POLE-mutated and double-classifier subgroups, short-to-moderate follow-up, limited event numbers, lack of centralized external pathology review, potential selection bias from surgically treated patients only, and lack of CTNNB1/L1CAM or other additional markers relevant to NSMP risk refinement were other major limitations of this study.

## 5 Conclusion

Molecular subgroups of endometrial cancer show distinct recurrence patterns. POLE ultra mutated patients have excellent outcomes while p53 abnormal patients faced worse outcomes. Molecular profiling tailors treatment to individual risk, allowing for optimization of therapy. This approach is a key step toward personalized treatment for endometrial cancer. Phased adoption of molecular testing can bridge the gap between precision oncology and resource constraints, ensuring equitable care for endometrial cancer patients in LMICs.

## Data Availability

The raw data supporting the conclusions of this article will be made available by the authors, without undue reservation.
